# An Open-Source Platform for Indoor Environment Monitoring with Participatory Comfort Sensing

**DOI:** 10.3390/s23010364

**Published:** 2022-12-29

**Authors:** Joseph Rosenberger, Zhong Guo, Austin Coffman, Duzgun Agdas, Prabir Barooah

**Affiliations:** Department of Mechanical & Aerospace Engineering, University of Florid, Gainesville, FL 32611, USA

**Keywords:** participatory sensing, comfort perception, open-source, wireless sensor network, indoor environment

## Abstract

We present an open-source wireless network and data management system for collecting and storing indoor environmental measurements and perceived comfort via participatory sensing in commercial buildings. The system, called a personal comfort and indoor environment measurement (PCIEM) platform, consists of several devices placed in office occupants’ work areas, a wireless network, and a remote database to store the data. Each device, called a PCFN (personal comfort feedback node), contains a touchscreen through which the occupant can provide feedback on their perceived comfort on-demand, and several sensors to collect environmental data. The platform is designed to be part of an indoor climate control system that can enable personalized comfort control in real-time. We describe the design, prototyping, and initial deployment of a small number of PCFNs in a commercial building. We also provide lessons learned from these steps. Application of the data collected from the PCFNs for modeling and real-time control will be reported in future work. We use hardware components that are commercial and off-the-shelf, and our software design is based on open-source tools that are freely and publicly available to enable repeatability.

## 1. Introduction

The primary purpose of HVAC control systems is to provide healthy and thermally comfortable indoor climates for occupants [1]. Thermal comfort is defined as “that state of mind which expresses satisfaction with the thermal environment” [2]. A key challenge in providing thermal comfort to building occupants is the lack of thermal comfort sensors. A host of factors affect a person’s perception of comfort [1]. For instance, the well known Fanger’s comfort index depends on variables, such as the metabolic rate and clothing insulation [3], and these variables are impossible to measure with current technology in a manner that does not disrupt an occupant’s normal activity.

Since an individual is the best sensor for what is comfortable to them, a climate control system should ideally involve the occupant in the climate control loop. This is the notion behind participatory sensing  [4,5]. The key challenge is to obtain useful information without disruptions to occupants (a recent increase in participatory sensing research is presented in Section 2). However, there is a dearth of work on developing indoor monitoring systems for office buildings that (1) can collect building occupants’ thermal comfort perceptions and environmental data from which predictions of comfort can be made later based on environmental data alone, and (2) can be part of an intelligent HVAC control system. To be part of a real-time control system, such systems must not be disruptive to occupants’ normal routines and they must be able to operate for long periods of time with little maintenance or repair.

**Figure 1:**
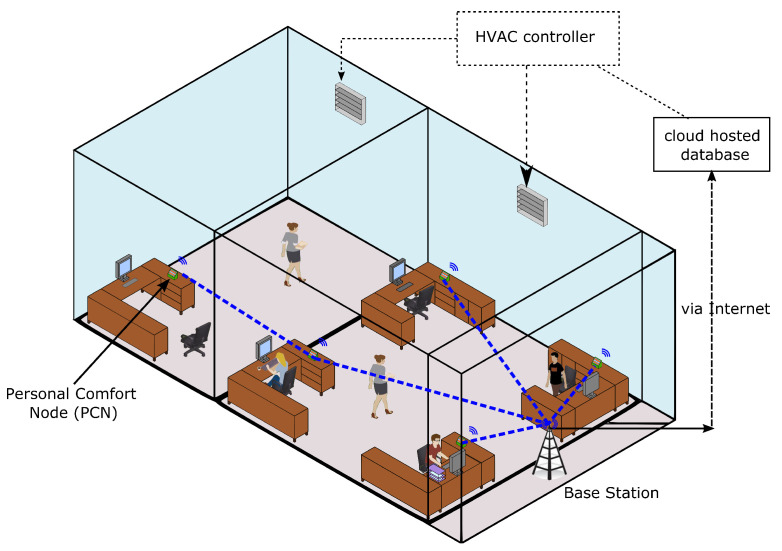
An overview of the PCIEM platform.

The *Personal Comfort and Indoor Environment Measurement (PCIEM)* platform described in this paper is designed to meet the two requirements mentioned above. The platform consists of a network of devices connected via a wireless network to a base station that pushes the data from these devices to a database via the internet. Each device is called a *Personal Comfort Feedback Node* (PCFN). Every PCFN has several sensors that measure environmental variables continuously. Every PCFN also has a touch screen through which users can provide feedback on their perceived comfort any time they wish, but otherwise, the PCFN does not disrupt the occupant in any way. The goal of the PCFN is to use both the regularly measured environmental variables and comfort perception feedback provided by the user to learn a *personalized comfort model* of the specific user that interacts with the PCFN. Eventually, an HVAC control algorithm will be able to use this model to predict what combination of environmental conditions will keep that particular individual comfortable, which can be used as part of an optimal control algorithm to achieve different objectives, e.g., minimizing energy use while keeping comfort within a range. Figure 1 illustrates how the PCIEM platform is expected to look when deployed in an office building.

In this paper, we describe the design of the *PCIEM platform*, which includes the PCFN devices, the network backbone (base station and the database structure), and the lessons learned during its design, prototyping, and deployment. The details of how the data from the PCFNs will be used to compute personalized comfort models, and how the models can be used for real-time control of an HVAC system, are beyond the scope of this paper.

We made all data needed to recreate the platform (including the software and hardware design files, server-side scripts, etc.) publicly available [6].

The choice of environmental sensors in the PCFN is dictated by the envisioned use of the data to create a “personalized comfort model” from the data, i.e., a mapping between the environmental sensor data and the recorded occupant discomfort level, without the use of disruptive/wearable sensors, such as heart rate monitors or skin patches. Since it is not clear at present what environmental measurements are needed to identify personalized comfort models, the PCFN is designed so that additional sensors can be easily added to it.

The rest of the paper is organized as follows. Section 2 details the related work and clarifies the difference with our work. Section 3 described the prototype of the system and its production version, while Section 4.3 reports on a preliminary deployment in a large office building. Section 5 describes the lesson learned during the development. The paper concludes with Section 6.

## 2. Related Work and Contributions

### 2.1. Related Work

There is a plethora of work describing wireless sensor networks for collecting indoor environment data [7,8,9,10,11]. These systems, however, do not collect comfort perception data from the occupants. They are, therefore, not suitable for personalized comfort modeling or HVAC control to improve comfort.

Works that are focused on constructing personalized comfort models typically use not only environmental sensors and a method to solicit perceived comfort feedback, but also comfort-related physiological measurements, such as skin temperature, heart rate, and acceleration [12,13,14,15]. Nkurikiyeyezu et al. [12] used a heart rate monitor to collect physiological data along with comfort surveys to construct a comfort model. Moreover, Dai et al. [13] attached thermocouples on several body parts of their experiment participants to collect skin temperature. Similarly, Liu et al. [14] used wearable skin temperature sensors (such as the iButton) and accelerometers as physiological sensors. Lastly, Quintana et al. [15] used a smartwatch—which provides heart rate and resting heart rate—along with wearable temperature sensors attached to the smartwatch to collect data from several occupants in three buildings.

The works above vary in the manner in which comfort perception data from occupants are collected: manually [12,13], through a web-based interface [14], via a smartphone app (e.g., [16]), or a smartwatch app (e.g., [15]). These systems were typically designed to aid high-quality data collection for comfort modeling but not for long-term deployment. For instance, participants in [14] were asked to complete surveys at least 12 times a day, for the study period of 14 days. Thus, while these systems can lead to valuable data for personalized comfort models, the resulting survey fatigue [17] renders them unsuitable to be part of a closed-loop HVAC control system. Moreover, the use of physiological sensors, such as skin temperature sensors attached to a smartwatch or heart rate monitors can be quite intrusive. Recent works have striven to reduce the level of intrusion. For instance, Li et al. [18] developed a system to infer skin temperatures with video cameras. The paper by Aryal and Becerik-Gerber [19] reported on laboratory experiments conducted with two human subjects. Physiological data—skin temperature data collected from a thermal camera and sensor attached to the participant’s wrists—combined with air temperature sensors improved accuracy over that using air temperature alone. However, the improvement is insignificant (3–4%), and may not justify the increase in cost and privacy concerns associated with physiological parameter monitoring.

The many advantages of using a smartphone or a smartwatch to obtain the users’ comfort feedback via an app comes with several significant challenges. One of them is to ensure that a particular person’s smartphone is mapped to the temperature sensor in the room that the person is occupying in a given time instance. In addition, some of the critical sensors needed for correlating comfort feedback to environmental conditions, such as space temperature and humidity, are lacking. Although most smartphones have sensors to measure the temperature of the processor or that of the interior of the phone, the measurement can be dramatically different from the space temperature the person occupies, especially if the smartphone’s apps are being used actively and create heat.

Pitt et al. [20] developed and deployed a network of nodes that have both environmental sensors, and means for collecting comfort feedback for constructing personalized comfort models without any intrusive sensing modalities. Each node has mechanical buttons to record “too hot”, “too cold”, and “just fine”. Each node also records several environmental sensors: temperature, humidity, several light levels, sound intensity, motion, and pressure.

There is another category involving participatory sensing that is primarily focused on the closed-loop control of HVAC systems to improve comfort, but comfort modeling is not their primary focus. One such commercial system is the Comfy App^TM^ (www.comfyapp.com), accessed on 2 April 2022. which uses smartphone apps for seeking comfort feedback from occupants. Gupta et al. [16] describe a system that provides an end-to-end system for controlling the HVAC system to provide personalized comfort. It requires occupants to specify the upper and lower limit of temperature they prefer via a smartphone app, which serves as the personalized comfort model. The same app also allows an occupant to provide their perceived comfort feedback (hot or cold), and this feedback is then used to change the comfort model if needed. Measurements of space temperatures from additional sensors are then used to infer the optimal temperature set point to minimize a deviation from the desired temperature range. Jazizadeh et al. [5] used a smartphone app to enable users to provide comfort feedback, which was combined with space temperature sensors to construct a temperature-based comfort model. Optimal setpoints for the HVAC system were computed based on this model.

Some works focused on HVAC control used wearable sensors in addition to environmental sensors. Feldmeier and Paradiso [21] described such a system with a network of wearable sensors. Users can provide feedback on comfort (hot, cold, or fine) through an interface on the wearable device. The devices also recorded the temperature, humidity, light level, and movement of the person wearing it via an inertial sensor. This information is used to change the HVAC system’s set points. Jung et al. [22] used a network of sensors that include smart wristbands worn by occupants to control indoor climate. The occupant activity using the acceleration data from the smart wristband is used as a surrogate for comfort.  Li et al. [23] reported on a system for closed-loop control to improve comfort. A personalized comfort model is constructed by data collected in offline experiments. Cheek temperature measurement is used during real-time control to predict perceived comfort, and this prediction is used by an optimizer to compute optimal setpoints for the HVAC system.

### 2.2. Motivation and Contribution

The motivation behind this work is to develop a participatory sensor network for office buildings—from which personalized comfort models can be constructed—with the following properties: (i) the sensor nodes are not disruptive to occupants, (ii) the network can be deployed easily and operated for long periods with little maintenance and repair, so that comfort models can be updated over time and the network can be part of an HVAC control system, and (iii) the network—both hardware and software—can be reproduced and improved upon by other researchers.

Recall from the review of related work that most studies on personalized comfort modeling use sensors that are disruptive to the normal functioning of occupants or have privacy issues. Some works-especially those that focus on HVAC control-have used less intrusive smartphones and smartwatches. However, as discussed above, the advantages offered by these smart devices are offset by serious limitations for comfort modeling and control. Due to requirement (i), the PCIEM platform we report on here eschews any kind of wearable sensor and smartphone/smartwatch. To avoid survey fatigue, we allow users to interact with the system as frequently or infrequently as they wish. Requirement (ii) made wireless communications with ad hoc networking—a feature of the PCIEM platform—essential.

The PCIEM platform we present in this paper is closest to that reported by  Pitt et al. [20]. The main difference is that our system uses wireless communication while that reported by Pitt et al. [20] used wired communication with Ethernet ports. Wired communication restricts where sensor nodes can be placed. Wireless communication in contrast makes it possible to deploy nodes at optimal locations for collecting environmental data and encouraging user interaction without intruding on them.

The third requirement is inspired by the open-source philosophy espoused by  Ali et al. [8]. To meet this requirement, all information needed to recreate the platform (the software and hardware design files, server-side scripts, etc.) are made publicly available [6]. The system described in [8] does not have a participatory sensing feature to collect comfort feedback and uses wired communication.

Apart from the differences between Pitt et al. [20] and Ali et al. [8], there are two additional differences between our work and all of the related work discussed so far. The first is a discussion on cost. Most papers do not report the cost of system development and those that do provide discussions and limit them to hardware costs. In practice, development (software) and maintenance (hardware and software) costs can be substantial and should be acknowledged as such. The second difference is that we discuss extensively the lessons learned in the development process, especially the mistakes made and their eventual remedies. We hope others who wish to develop such a system and improve upon it can benefit from these discussions.

## 3. The PCIEM Platform: Prototype

The PCIEM platform refers to a collection of PCFNs, a base station, and a database server. The individual PCFNs collect indoor environment measurements from sensors and perceived comfort feedback from the occupants. The base station is responsible for collecting the data from all of the PCFNs and exporting them the database server. The database server is hosted in a remote computer, though in principle it can reside in the base station itself.

### 3.1. The PCFN

The PCFN is equipped with a capacitive touchscreen, several environmental sensors, a microprocessor, a radio, and a power supply. The radio transmits the data from the PCFN to the base station every 10 s, except when the user interacts with the PCFN, which will be described below. Although environmental variables do not change frequently, the 10 s duration was chosen in order to provide robustness to data loss from random transmission failure. In addition, the presence of the touchscreen meant the PCFN needed wall power, so the additional energy use due to frequent transmission is not a concern.

The capacitive touchscreen is the key component that allows an office occupant to provide comfort feedback. The touchscreen is programmed to display a comfort perception scale, −3 to 3, from extremely cold to extremely hot. The numerical value corresponding to the slider is indicated on the touchscreen. The user can move the bar in the middle of this scale to indicate their perceived comfort within the range. As the user moves the bar, the display of the corresponding numerical value on the touchscreen is updated. A software-defined “update” button is placed on the capacitive touch screen. When the user presses the “update” button on the touchscreen, the sensor data and the comfort feedback are immediately polled and transmitted to the base station.

Measuring perceived thermal comfort and designing a user interface to collect feedback are complex problems. Several, distinct thermal comfort sensation scales have been proposed in the literature for assessing occupants’ perceptions [5]. Our choices of the comfort scale and the user interface are made based on a trade-off between ease of use and the fidelity of the feedback.

The choice of environmental sensors is dictated by the eventual goal of identifying a comfort model from the data, i.e., a mapping of environmental measurements to occupant’s perceived thermal comfort. Because of the uncertainty about the environmental variables, except for temperature and humidity, we decided to add the following sensors: (i) Air temperature sensor, (ii) air humidity sensor, (iii) CO${}_{2}$ concentration sensor, (iv) VOC (volatile organic compound) concentration sensor, (v) light level sensor, and a (vi) PIR motion detector (to measure occupant presence).

A DS18B20 digital thermometer was used as the ambient temperature sensor. It has an advertised resolution of ± 0.5 °C, does not require a separate power supply, and can draw the necessary power from the data line. Humidity is measured with the HIH-4030 sensor, which has an accuracy of ±(5to8)%. It needs a 4–6 volt power supply, which is within the range of feasible voltages for the overall PCFN. A SenseAir K-30 1% sensor was chosen for the CO${}_{2}$ concentration measurement due to its widespread use in environmental monitoring. A Parallax PIR sensor was used for motion detection. We refer the interested reader to  Ali et al. [8] for a detailed description of these two sensors and their underlying technologies. A PDV-P8103 photocell was used for measuring light intensity. Since it was not clear how much effect light intensity will have on an occupant’s thermal comfort perception, if at all, light intensity sensing was not considered critical. The PDV-P8103 is an inexpensive and extremely simple sensor—a photosensitive resistor. So the sensor has to be calibrated by the user if its reading is to be translated to lumens. Similarly, a VOC sensor (MiCS-5524 from SGX Sensortech) was added to the PCFN to enable the measurements of pollutants other than CO${}_{2}$  which might be correlated with poor indoor air quality and perception of comfort. This sensor can detect many types of volatile organic compounds, such as CO and ammonia, but as with the case of light, the scalar reading of the sensor does not provide high-resolution information about any specific gas. As with light, the VOC concentration measurement was considered non-critical; thus, more expensive options were not considered.

The brain of each PCFN is an Arduino Mega 2560, which was chosen to adequately support the devices attached to it (sensors and touchscreen). Due to the libraries required to support the capacitive touchscreen, lighter and cheaper options, such as the Arduino Uno, were eliminated. The Arduino Mega also has more I/O pins, so the design is robust to the future demands of more sensors.

The components of the PCFN are powered by a 9 V DC power supply that takes input power from a 110 V single-phase AC wall outlet. It is rated for the 1A DC supply and is needed because of the high power demand of the touchscreen. Note that the high power and energy demand of the touchscreen that eliminates the battery is a possible source of power. An XBee Pro 2.4 GHz radio was used, together with an RP-SMA antenna for extending the range of wireless transmission.

#### Sensor Characterization

Most of the sensors used in the PCFN were low-cost hobby-grade sensors except the CO${}_{2}$ sensor, which makes its accuracy and reliability a concern. The K-30 CO${}_{2}$ sensor is widely used, and a comparison of its measurements with another CO${}_{2}$ sensor is provided by  Ali et al. [8]. Similarly, a comparison of the measurements from the Parallax PIR motion detector was also provided by  Ali et al. [8]. So we do not characterize the K-30 and the PIR motion sensors here.

Among the remaining measured variables, temperature and relative humidity are expected to be critical for comfort modeling, so we characterized the sensors for accuracy and consistency before integrating them into the PCFN. Data were collected from ten distinct temperature and RH sensors (with the same part numbers; they were purchased together) that were placed on a desk in physical proximity, before being integrated into PCFNs. The ground truth for the temperature and humidity sensors is a Vaisala HM70 humidity and temperature sensor that is accurate up to ±1% relative humidity. The temperature sensors are consistently accurate. However, the humidity sensors are less accurate, and there is a bias among the sensors.

Each PCFN is assigned a unique identifier, called the UID (or uid) in the following. This information is embedded into the Arduino code while programming the PCFN. Each data packet has the UID of the transmitting PCFN in it, which is forwarded by the *base station* to the *database server*.

### 3.2. The Base Station

**Figure 2:**
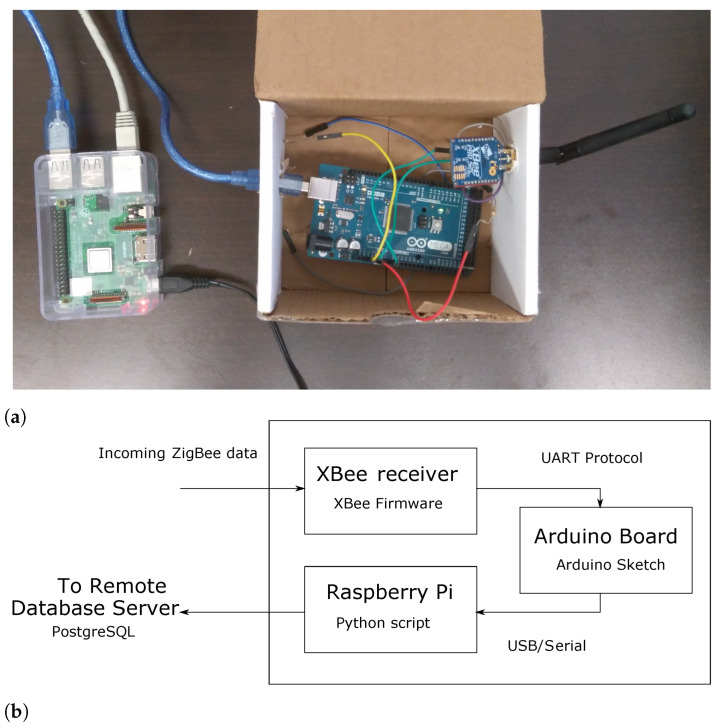
The base station (prototype version) and data flow. (**a**) A prototype of the base station. (Left): Raspberry Pi, (Right): Arduino and Xbee Pro^©^ receiver; (**b**) data flow inside the base station.

The base station has two functions: (i) receive data packets from the PCFNs, and (ii) push these data packets, after time-stamping them, into a remote database server. The base station consists of two main pieces of hardware: a wireless receiver, and a general-purpose computer with an internet connection; see Figure 2.

ZigBEE was chosen as the wireless communication protocol. The communication transfer requirement (in bytes) is low and the envisioned number of devices is at most a few hundred for a single building, typically less. These requirements make ZigBEE more favorable as compared to Bluetooth or WiFi [24]. Another critical advantage of ZigBEE is that is an open protocol and capable of automatic mesh networking, so that data from a PCFN device that is not in direct range of the base station is automatically routed to the base station via other PCFNs.

**Figure 3:**
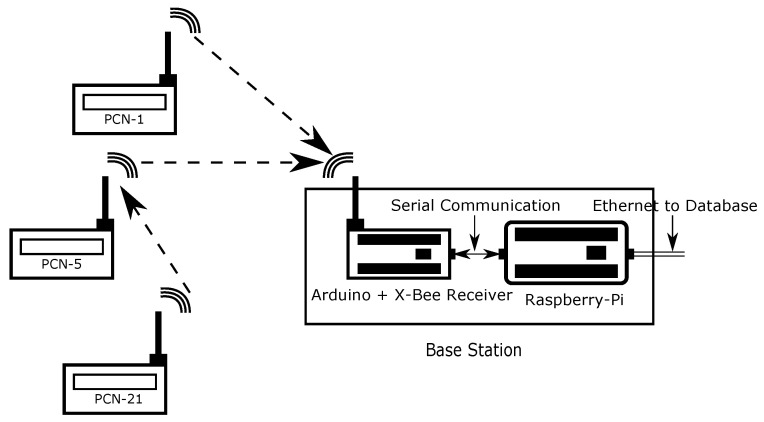
A schematic of the data transfer chain over the PCIEM network.

The PCIEM network described here is comprised of two types of members: (i) the transmitters, PCFN’s (“Router” in ZigBEE mesh terminology) and (ii) a single receiver in the Base Station (“Coordinator” in ZigBEE mesh terminology) (see Figure 3). Just as the PCFN transmitters, the receiver in the base station also uses an XBee radio. The pipeline for data flow from PCFNs to the remote database server through the base station is illustrated in Figure 3.

The wireless receiver in the base station is an Arduino Mega with an Xbee Pro${}^{©}$ radio, the same as that in PCFNs. A Raspberry Pi model 3B+ was selected as the computer, which runs a Linux-based operating system. The Pi has internet connectivity via both Ethernet and WiFi.

The Xbee Pro${}^{©}$ is connected as the only peripheral to the Arduino, and it is powered from the 3.3 V Power Pin on the Arduino. The UART (Universal Asynchronous Receiver/Transmitter) interface is utilized for data transfer from the Xbee Pro${}^{©}$ to the Arduino. The Arduino board is connected to Raspberry Pi through a USB cable, which simultaneously powers the Arduino board and facilitates serial communication between Raspberry Pi and the Arduino board using an FTDI chip. Raspberry Pi is powered by its own power supply.

The entirety of the software utilized in the base station and database server is developed using open-source tools. A Python script running on Raspberry Pi pulls data received from the Xbee Pro${}^{©}$ radio (receiver) through the Arduino microprocessor (via the USB/serial connection) to Raspberry Pi (see Figure 2b). The same Python script also time-stamps the data and pushes it to the remote database through the Internet. Since the base station (rather, the Raspberry Pi) is connected to the Internet and its clock is synchronized to a global clock, time stamps made at the base station are considered accurate. The only time inaccuracy comes from the delay between transmission from a PCFN and reception by the receiver Xbee Pro${}^{©}$ at the base station, which is small. It should be emphasized that a small timing error, smaller than a second, is negligible because of the intended applications: HVAC control and occupant thermal perception, which are dominated by processes with much slower time scales.

### 3.3. Database Server

PostgreSQL was chosen as the relational database management system since it is free, open-source, widely used, and has proved useful in our past work in managing large volumes of time-series data related to HVAC monitoring and control [25]. The database was designed to have only one table, with columns for uid, date-time, temperature, humidity, voc, co2, light, motion, and comfort. Each row of the table corresponds to the data collected from one PCFN at one time instance, with the column uid indicating which PCFN it is, the date–time indicating the time instances the data were received by the base station, and the rest being the sensor measurements. The Python library psycopg2 makes connecting to a PostgreSQL database server and pushing data into a database seamless. The database can be hosted anywhere—we used a desktop Linux machine running a postgreSQL database server.

### 3.4. Automatic Restart on Power Cycle

Ensuring automatic restart of the data collection and transfer in the event of a power cycle is essential in achieving the goal of low maintenance. A power cycle refers to the loss of electricity supply to one or many of the hardware components, followed by restoration. When power is restored, the end-to-end data transfer should resume without requiring any human intervention, especially on the PCFNs or the base station, otherwise, the maintenance cost of the network will be extremely high. This lesson was learned the hard way in a previous project, in which a wireless sensor network was developed and deployed in a building that did not have an automatic restart capability [26,27]. Although the network was successfully used for closed-loop HVAC control (see Brooks et al. [25]), maintaining the network required manual labor due to the occasional and temporary loss of power supply to the base station that occurred.

The receiver Xbee Pro${}^{©}$ in the base station was robust to such a power cycle since the Arduino processor restarts executing its embedded code whenever a power cycle occurs. The same is true for the PCFNs but some care is needed to ensure the Python script in Raspberry Pi of the base station restarts after a power cycle and successfully reestablishes the data transfer process. Because Raspberry Pi runs on a Linux stack, many methods are available for such automation. We tested several methods; more than one worked. However, some were less reliable and more complex than others. The method we finally chose requires adding a single line to the autostart file that is already part of the base Raspbian installation (or any other standard Linux distribution). The location of the file may vary depending on the distribution, but in the Raspberry Pi we used, the file was located in /etc/xdg/lxsession/LXDE-pi. The following line has to be added to the end of the autostart file in that folder: /usr/bin/python2.7 <path to python script>, where “python script” refers to the one that pulls data from the XBee receiver and pushes to the remote database.

We found that no special design is needed on the database server side to enable the automatic restart of data collection. Even if the database connection is lost due to the powering down of the base station, once the python script at the base station restarts, it is able to reconnect to the database server and push data.

## 4. The PCIEM platform: Production Version, Cost, and Deployment

In this section, we discuss an improved version of the PCFNs (over the prototype) to make it more suitable for deployment and long-term use in a real office building, the cost incurred in developing the end-to-end PCIEM platform, and experience from the deployment of the PCIEM platform in a real building, including the lessons learned.

### 4.1. Production Version of PCFNs

During prototype development, various components in both the PCFN and the base station, such as the Arduino Mega, sensors, and radios were connected with jumper wires. Such a design has a high probability of failure over long time periods with wires becoming loose. Moreover, many of the sensors came with additional peripherals when purchased that were not only unnecessary but also added bulk and power consumption. Therefore, once the prototype of the platform was tested and verified, we redesigned and fabricated the PCFN and the base station for greater reliability through an electronic design and fabrication company: Out Of The Box Robotics (oobrobotics.com), in Gainesville, Florida, USA, who will be referred to as the vendor in the following. The resulting system is termed the *production version*, to distinguish it from the prototype version.

**Figure 4:**
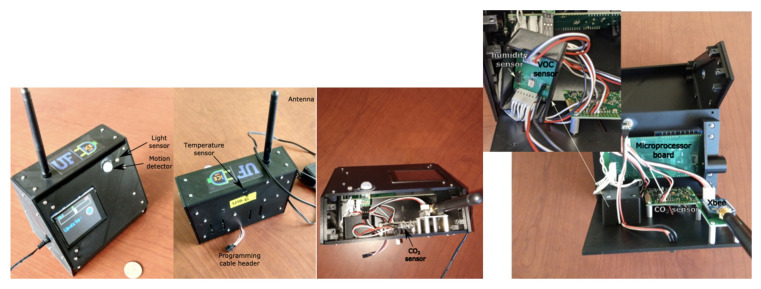
A production version of a PCFN with the internals exposed. The slots on the back cover and the gap on the front cover ensure adequate airflow through the case for the VOC and humidity sensors.

A production version PCFN device is shown in Figure 4. Instead of using the entire Arduino Mega board and the sensors with their breakout boards, the main processor of the Arduino, and the main components of the sensors were used in a printed circuit board (PCB) that was custom designed by the vendor. This version was also more convenient for assembly and disassembly. In the case of the base station, only the receiver node (radio and microprocessor) changed, and the computer stayed the same (Raspberry Pi).

Except for the professional redesign of the circuit boards and assembly of the PCFNs, everything else was the same between the prototype version and the production versions with the exception of the location of the temperature sensor. The heat radiated by the touchscreen is sufficient to raise the interior temperature of the PCFN case to create a biased temperature measurement, should the sensor be placed in the case without any special protection. Initially, we attempted to negate this effect by designing the plastic case of the PCFN to include a plastic separator between the touchscreen and the temperature sensor and adding as many slots as possible in the case for airflow. While this design appeared to be successful initially, subsequent testing created doubt about the reliability of the temperature measurement. So, finally, the PCFN and the circuit board were redesigned so that the temperature and humidity sensor stick out of the back of the case, exposed to the environment they are supposed to measure; see Figure 4. This is a suboptimal design since the temperature sensor can be inadvertently damaged by the user; it no longer benefits from the protection provided by the case. Still, we proceeded with this design for the production version in the interest of measurement accuracy.

### 4.2. Cost of the PCIEM Platform

A detailed cost breakdown of the components of the PCFN (production version), including the assembly cost charged by the vendor, is provided in Table 1.

The cost of the prototype PCFN was around USD 284, slightly lower than the cost the production version (USD 331). Most of the difference was due to the USD 100 charged by the vendor for manufacturing, which was not incurred in the prototype. It should be noted that labor costs were not included in the cost estimate of the prototype PCFN. The prototype did incur additional costs in the hardware since we used the full Arduino Mega development board (around USD 45) instead of simply using the microprocessor, and the case for the prototype was 3D printed.

In addition to the PCFNs, a Raspberry Pi computer with an XBee receiver board was used as hardware for the base station, with a combined cost of around USD 100. The database was hosted on a remote computer, so in principle, one could include the cost of that computer as well. However, the desktop Linux computer we used as the database server was already being used for other purposes, so we did not count its cost as part of the PCIEM platform. If this system is used commercially, it makes more sense to host the database in the cloud. In that case, one has to include cloud server costs as recurring operating costs.

A significant part of the development effort was incurred in designing and testing the PCFNs, in writing and testing the scripts that run in the base station, and then testing the PCIEM platform to ensure reliable data collection, transmission, and reception. Because that effort was incurred by undergraduate and graduate researchers as part of their education, the time spent is not indicative of what will be needed from an experienced professional. A rough estimate of the development time is four human months for an experienced embedded systems developer and one human month for an experienced software developer. If the designs, codes, and lessons learned from this project are utilized to recreate the system, the required effort and cost will be lower. If further improvements over this system are sought, additional effort will be needed.

### 4.3. Deployment in an Office Building

The University of Florida’s *Innovation Hub* (iHub) was chosen as the demonstration site. A PCFN platform with the production version of the PCFNs and the base station was deployed in iHub in April 2021. The database was hosted on the same Linux machine that was used during prototyping.

Due to COVID-19-induced delays and a preference for remote work that reduced the number of regular office occupants in the building, we were able to recruit only a small number of volunteers to be part of the study. So we started with a much smaller deployment than planned, consisting of only five PCFNs. An analysis of the data from this small network still provides useful information on the functioning and performance of the platform in a realistic setting since the nodes of the network spanned three floors and a floor space of approximately 50,000 sq. ft.

**Figure 5:**
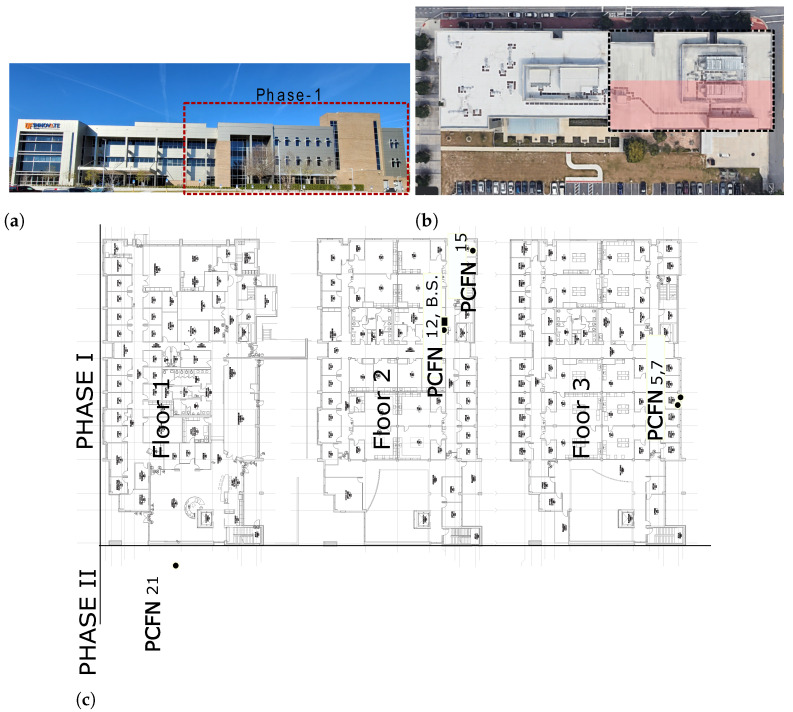
The deployment site, iHub (innovation hub), located at the University of Florida campus, and locations of PCFNs inside iHub. (**a**) Picture of the Innovation Hub building (view from south to north). Phase-1 is enclosed in the dashed lines; (**b**) top view of iHub. All but one of the PCFNs are deployed in the southern half of Phase-1 (shaded in blue)—served by an air handling unit (AHU-2). Imagery ©2021 Maxar technologies, U.S. geological survey, Map data ©2021 Google, maps.google.com (22 January 2021); (**c**) locations of the PCFNs deployed in iHub and that of the base station (marked as B.S.).

**Figure 6:**
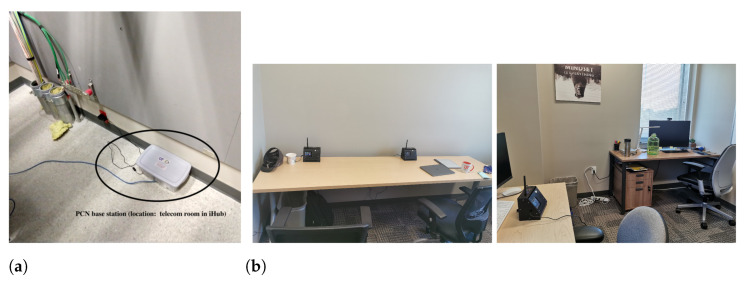
Base station and a few PCFNs, after deployment in iHub. (**a**) Base station in iHub; (**b**) a few PCFNs, as installed in offices in the iHub building.

Figure 5 shows the building. The locations of the PCFNs in the building are shown in Figure 5a. The base station is shown in Figure 6a, which was installed in an unoccupied room that houses communication equipment, allowing access to an Ethernet port without disruption or raising concerns about clutter. The base station hardware, including the radio and antenna, is inside the plastic box for protection from the environment.

Photographs of a few PCFNs installed in office occupant’s workstations are shown in Figure 6b. PCFN 21 is in a room in Phase II—a recent extension to the building, while the other PCFNs are in Phase I of the building. Note that even though the number of PCFNs deployed is small, they are spread over all three floors of the building, making them quite far apart from each other. Still, as we will see in the sequel, data communication was reliable.

### 4.4. Data from the PCIEM Platform

Data were collected from PCFNs from April 2021 to September 2021, providing a large enough window to assess system performance and reliability. Data collections from the PCNs were stopped in October 2021 due to a lack of funding to continue the project, especially since a larger number of devices needed to be deployed for the next phase of the project: constructing personalized comfort models from data. Still, the deployment from April to September provided a useful window into the performance and reliability of the platform.

**Figure 7:**
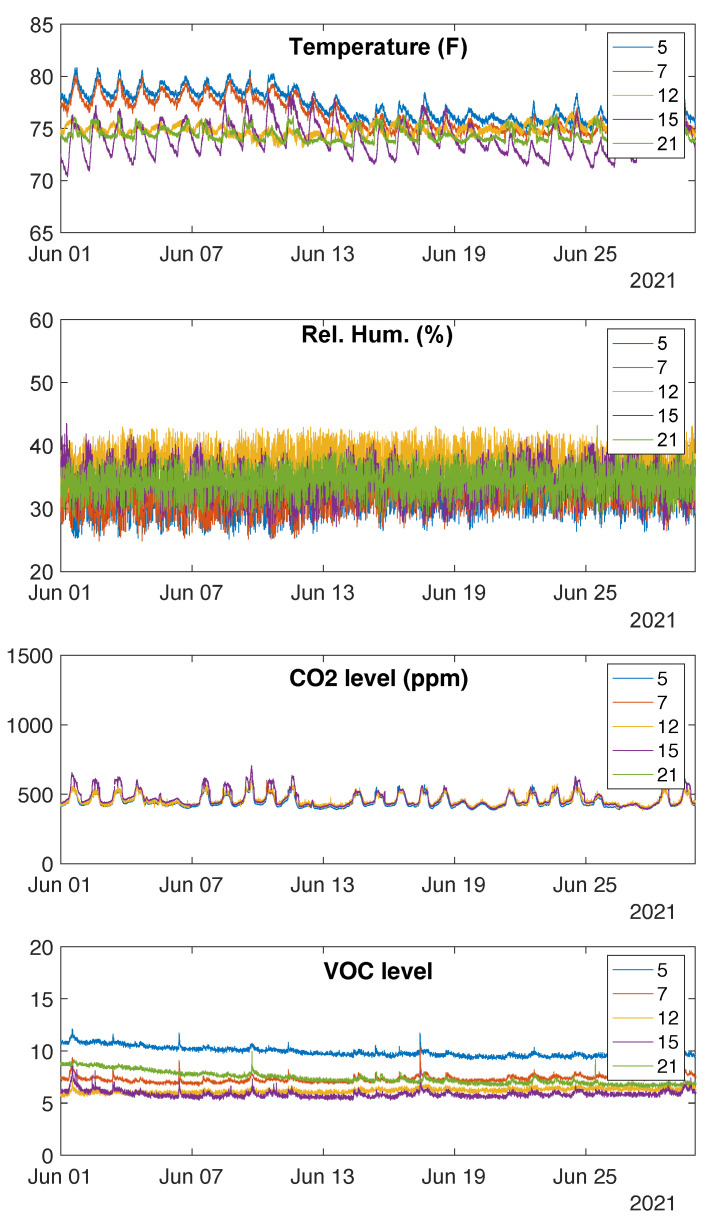
Data on four environmental variables collected by sensors in the PCFNs deployed in the iHub. Legends correspond to UIDs of PCFNs.

The PCFNs transmit data every 10 s, except for those instances when the user presses the “update” button. More than 99% of the samples were received with an inter-sample interval of 10 s, showing highly successful data transmission and reception despite the spatial variability of the deployments. A time series of four environmental variables collected from the PCFNs for the month of June 2021 is shown in Figure 7.

**Figure 8:**
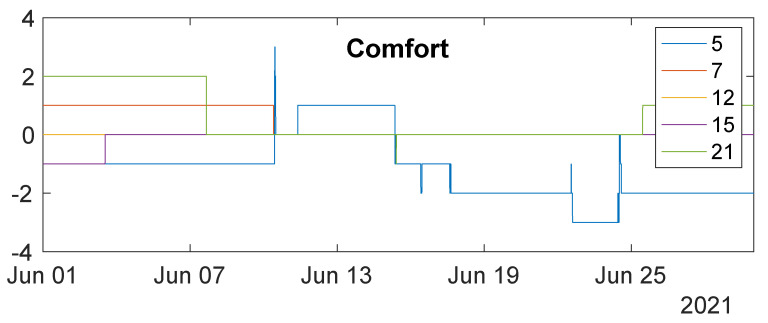
Comfort feedback recorded by PCFNs deployed in iHub over a month.

The comfort perception feedback provided by the corresponding users is shown in Figure 8. Occupants only intermittently interacted with the PCFNs. The user of PCFN 5 has provided the most amount of feedback during the period, and this user’s comfort has varied and fluctuated over time, between quite hot to quite cold. That is consistent with the environmental measurements shown in Figure 7: this PCFN has seen some of the largest (and most) variations in the indoor climate among those recorded. Similar fluctuations in temperature are also present for PCFN 7, but this user provided limited feedback. This difference may be attributed to the differences among users’ personalities or thermal comfort perceptions. Due to the small sample size, not much more can be said at this stage. Since PCFN 12 was installed in an unoccupied room, there is no occupant interaction; so its comfort value always remained at 0.

## 5. Lessons Learned

We list the lessons learned during the development process below.

1 Overall, obtaining a functioning prototype of a single PCFN was straightforward. However, obtaining the wireless communication aspect of the PCIEM platform working reliably was much more challenging. Programming the Xbee Pro${}^{©}$ radios is not trivial. Multiple radios and programming boards tried to eliminate hardware issues. Many radios with lower costs are even easier to use, but we chose Xbee Pro${}^{©}$ for the simple reason that these radios have been consistently available for many years and are unlikely to disappear in the near future. In the do-it-yourself (DIY) ecosystem of development boards, sensors, and radios, obsolescence is common and frequent. In fact, the company that made one of the radios we tested at the beginning of the project went out of business during the course of the project, making it impossible to buy more radios of that type.
2 As discussed in Section 4.1, the heat generated by the touchscreen was a concern for the temperature sensor. This was resolved by putting the sensor outside the case, but in the future, a more elegant solution will be preferable. In any case, the design of the housing is important to ensure a high degree of airflow into the case, since otherwise the VOC and CO${}_{2}$ sensors readings will be different from what they are meant to measure: concentrations in the ambient near the PCFN.
3 The current rating of the power supply is important. Because the touchscreen draws considerable power when its display was on, and together with the power draw of the other components, the combined demand can be higher than what a lower-rated 9V power supply can deliver. In such a scenario, the sensors will produce biased readings. This issue was discovered during the prototyping stage when a lower-rated power supply was used.
4 The software for the PCFN was initially written to send the same data packet up to ten times until an acknowledgment (ACK) was received from the receiver at the base station. It was discovered during a network test with many PCFNs that after a few days, the PCFNs stopped sending data. When the “repeat until ACK received feature” was removed from the transmitters, the problem vanished. Although the reason is not quite clear, data transfer was highly reliable even without this feature as reported in Section 4.3, so the feature was removed in the production version.
5 Cyber-security is a potential issue due to the fact that a general-purpose computer (the Raspberry Pi) is part of the base station and is connected to the Internet constantly, while being unattended. These concerns were ameliorated by not keeping a monitor/keyboard/mouse connected to the base station and setting up screen lock and password for the Raspberry Pi.
6 Another lesson learned from the project was the value of ZigBEE mesh networking. A wireless sensor network was developed by our team in a past project for real-time indoor climate control that did not use ZigBEE. The details of the network are described in the two MS theses [26,27]. The network was developed to enable closed-loop HVAC control, and the resulting closed-loop experiments are described in Brooks et al. [25]. Two important lessons were learned in this earlier project; (i) it is important to avoid any proprietary tools in the development, and (ii) ad hoc mesh networking is critical for scalable deployment of a large indoor sensor network. The earlier design used a radio with a proprietary communication protocol (SimpliciTI${}^{\mathrm{TM}}$ [28]), which used a star communication topology, meaning that each transmitter had to be in direct range of a receiver. However, indoor spaces are challenging for radio communications, and sometimes two points that are close in Euclidean distance may still be out of range. As a result, range extenders had to be established after an initial deployment indicated the presence of wireless dark spots [27]. The use of mesh networking in this project eliminated that issue and reduced the time needed for deployment tremendously. In fact, the system described here had only one base station in a large building. Yet, it was still able to collect data from all of the PCFNs in the building due to multi-hopping, perhaps aided by the RP-SMA antenna that increased the range. Additionally, with an open protocol with a large user base, many resources are freely available to the developer. That was not the case for the proprietary SimpliciTI${}^{\mathrm{TM}}$ protocol, which too made development in the earlier project challenging.
7 Automatic restart of the entire system after a power cycle is critical.

## 6. Conclusions

We presented the design and preliminary deployment of a Personal Comfort and Indoor Environment Measurement (PCIEM) platform, which collects indoor environmental measurements and enables office occupants to provide feedback on their perceived comfort without any disruption to their normal routines. Building occupants interact with the PCIEM platform through an individual PCFN that is meant to remain in their work areas. The PCFN is equipped with a capacitive touch screen, so interacting with one is similar to that with a smartphone. Wireless networking with open protocols enables ease of deployment and maintenance.

All of the software, hardware design files, and installation instructions of the PCIEM platform are publicly available in [6] so that other researchers can reproduce the system and refine it. The platform is designed with free and open-source tools and off-the-shelf components to aid in such efforts.

Actual deployment in an occupied office building was hampered by the COVID-19 pandemic. We hope to perform a larger-scale deployment in the future. It will be particularly interesting to see how often users interact with the PCFNs to provide comfort feedback and how challenging it is to identify comfort models for participants from the data. There are many additional avenues for future research on the platform itself, such as reducing costs and sizes.

The platform is envisioned to aid in identifying personalized comfort models for individual office occupants, keep those models updated, and eventually be a part of a closed-loop HVAC control system. This paper only describes the development and deployment experience of the platform; not its use. The first future research task will be to use the data collected from the PCIEM platform to construct personalized comfort models. Following reliable personalized comfort model development, the next task will involve the use of such models in controlling HVAC systems to improve the occupant’s comfort.

## Figures and Tables

**Table 1:** Cost of PCFN (production version).

Parts	Description	
**Sensors**	**Part Name/Description**	**Price** **in $**
VOC Sensor	MICS-5524	11.96
CO${}_{2}$ Sensor	K-30	85
Motion detector	Parallax PIR	4.86
Light Sensor	DigiKey PDV-P8103	0.65
Humidity Sensor	HIH-4030-003	5.9
Temperature Sensor	DS18B20+	3.98
**Hardware**		
Capacitive Touch Screen	Adafruit 2.8” TFT	40.46
Microprocessor	ATMEGA2560-16AU	14.28
XBEE-Pro Radio 2.4 GHz	XBP24CZ7SIT-004	32.56
Duck Antenna	A24-HASM-450	5.5
Voltage Supply	DigiKey L6R12H-090	6.3
PCBs	–	15
Miscellaneous	screws, jumpers, housing	20
Assembly cost	-	100
**Total**		331.4

Prices in USD, in 2019–2020 dollars.

## Data Availability

The data presented in this study are available on request from the corresponding author. The data are not publicly available due to privacy reasons.

## Abbreviations

The following abbreviations are used in this manuscript:

PCFN	personal comfort feedback node
PCIEM	personal comfort and indoor environment measurement
HVAC	heating, ventilation, and air conditioning
